# “Try until the last?”—the relevance of fresh embryo transfer outcomes for subsequent same-cohort vitrified–warmed cycles

**DOI:** 10.1007/s10815-024-03285-5

**Published:** 2024-10-11

**Authors:** Julia Lastinger, Sabine Enengl, Peter Oppelt, Philip Sebastian Trautner, Thomas Ebner, Omar Shebl

**Affiliations:** Department of Gynecology, Obstetrics and Gynecological Endocrinology, Kepler University Hospital, Johannes Kepler University, Altenberger Strasse 69, 4040 Linz, Austria

**Keywords:** Cryopreservation, Embryo cohort, Pregnancy rate, Live birth rate, Prediction model

## Abstract

**Purpose:**

Taking into consideration earlier attempts at assisted reproduction and previous pregnancies allows a more differentiated approach when counseling couples regarding their chances in assisted reproductive treatments. The aim of this study was to investigate whether the outcome of fresh embryo transfers affects subsequent same-cohort vitrified–warmed cycles.

**Methods:**

This retrospective cohort study analyzes the outcome of all fresh and frozen embryo transfers (*n* = 8118) between January 1, 2011, and December 31, 2022. All women who received fresh or frozen embryo transfers at Kepler University Hospital Linz were included. The use of donor eggs, previously cryopreserved oocytes, and freeze-all cycles were excluded from the analysis.

**Results:**

Positive serum βhCG after a fresh cycle has a significant impact on the chances of positive βhCG in a subsequent vitrified–warmed cycle (36.3% [33.2%, 39.5%]; *P* = 0.002). Negative βhCG after a fresh cycle does not predict the outcome of the following same-cohort embryo transfer (33.1% [30.7%, 35.7%], *P* = 0.726). Pregnancy rates of the first, second, third, and fourth same-cohort vitrified–warmed embryo transfer remain continuously high, with 32.8%, 30.8%, 28.9%, and 27.1%, respectively.

**Conclusions:**

Positive serum βhCG after a fresh embryo transfer has a positive predictive value for the outcome of a subsequent vitrified–warmed embryo transfer. Couples with a negative fresh cycle should not be discouraged from continuing fertility treatment using same-cohort frozen embryos.

**Trial registration:**

This study was prospectively registered at the German Clinical Trials Register on September 5, 2023 (DRKS00032552).

## Introduction

Despite substantial advances in assisted reproductive technology (ART) in the last decade, the relatively low effectiveness of human reproduction makes it essential to continuously pursue improvement of ART outcomes. Various factors play a role in the success of fresh embryo transfers (ETs) and frozen embryo transfers (FETs), some of which have yet to be confirmed by clinical studies. Apart from individual female and male factors, researchers have comprehensively studied various determinants of successful ART, including oocyte and embryo quality [1, 2], day of embryo transfer [3–6], and ovarian stimulation protocols [7–9].

In some studies, a smaller number of oocytes acquired via mild ovarian stimulation were found to result in higher pregnancy rates [10]. This effect is probably because a homogeneous cohort of good-quality oocytes is retrieved. It has been suggested that larger numbers of oocytes may be associated with lower oocyte quality and subsequent lower embryo quality and that ovarian hyperstimulation may have a negative impact on endometrial receptivity [1, 10, 11]. Even though severe ovarian hyperstimulation syndrome occurs rarely, fatal cases have been described, mostly due to thromboembolic events. The mortality rate has been estimated between 1 in 45,000 and 1 in 500,000 [12, 13]. Obtaining one or more top-quality embryos strongly correlates with higher pregnancy rates, whereas low embryo quality is linked to significantly lower live birth rates [2, 14].

The improvement of laboratory technologies in ART and the latest recommendations for performing elective single embryo transfers have led to a larger number of supernumerary embryos being cryopreserved [15]. Consequently, ART cycles using previously cryopreserved embryos have become more common. Studies on live birth rates after ET and FET in normal responders have shown comparable results [8, 16, 17].

Couples with a long history of infertility have often undergone multiple fresh cycles and frozen–thawed embryo transfers. During this process, these patients often enquire whether they should attempt another ovarian stimulation or make use of their previously cryopreserved embryos. This dilemma is often associated with increased emotional stress in infertile couples [18]. For physicians counseling these couples, the question of which option will lead to the highest probability of pregnancy is difficult to answer.

There has been little research on the effect of the individual patient history—including previous in vitro fertilization (IVF) cycles, pregnancies, and live births—on the success of ART. Only small studies with conflicting results are available on the topic. Some research groups have claimed that achieving a live birth in a fresh cycle does not predict the outcome of same-cohort frozen cycles [19–21]. Others have argued that a successful pregnancy after the first fresh cycle correlates with higher pregnancy rates in same-cohort FET [7, 22–24]. One research group reported that couples with a negative ET have a higher chance of clinical pregnancy and live birth in the subsequent vitrified–warmed cycle when embryos from the same cohort are transferred [25]. Most of these studies only analyzed the first FET after a fresh cycle [19, 20, 22, 23, 25]. In other studies, no differentiation was made between women who were having their first same-cohort vitrified–warmed cycle and those who had had unsuccessful FET cycles from the same cohort before [7, 21, 24]. To the best of our knowledge, no data on multiple consecutive same-cohort FET outcomes have yet been published. In addition, only the slow-freezing method was used in many of the studies mentioned [7, 19, 22, 25]. For cleavage-stage embryos and blastocysts, vitrification has been found to be the most successful approach [26, 27]. One study group concluded that women with a first-trimester miscarriage after FET have a higher chance of a live birth in the subsequent FET [28].

The underlying physiological mechanisms leading to these findings remain unclear. Some researchers have hypothesized that there are changes in the endometrium leading to altered endometrial receptivity, while others have suggested that the optimal embryo for transfer is not always chosen correctly [3, 25, 28].

The aim of the present study was to evaluate whether there are differences in pregnancy and live birth rates when the outcome of previous same-cohort embryo transfers is taken into account. It also attempts to calculate individual pregnancy rates for each embryo transfer after consecutively transferring one fresh embryo and one, two, three, or more frozen–thawed embryos from the same cohort. The goal is to establish an approach that is both evidence-based and individualized for counseling infertile couples undergoing ART.

## Materials and methods

All fresh and frozen embryo transfers carried out between January 1, 2011, and December 31, 2022, in the Department of Gynecological Endocrinology at Kepler University Hospital Linz were included in this study. The patients were 18 years of age or older, with a mean age of 33.7 ± 4.7 years (range 18–47).

Embryos at cleavage stage were scored in accordance with the Istanbul Consensus [29], while blastocysts were assessed in accordance with Gardner et al. [30], focusing on expansion and the quality of both the inner cell mass and trophectoderm. Routinely, only day 5 embryos were cryopreserved in this study, using a previously published vitrification method [31]. In other words, in cases of cleavage-stage transfers, all surplus embryos were cultured up to day 5. Only blastocysts with good- to fair-quality cell lineages (≥ BB) were considered for cryostorage.

Cases of donor eggs, IVF and ICSI cycles with previously cryopreserved oocytes, and all stimulation cycles with a freeze-all regimen were excluded. IVF or ICSI was performed, depending on sperm quality, on the day of oocyte retrieval.

Outcomes of fresh embryo transfers and their associated frozen transfers from the same cohort were compared. Successful embryo transfer was defined as positive serum βhCG 14 days after the transfer date. Clinical pregnancy was defined as ultrasound visualization of a fetal heartbeat 28 days after embryo transfer.

### Ethical approval

The study was approved by the institutional ethics committee of Johannes Kepler University, Linz, Austria (EK 1178–2023, date of first approval August 24, 2023), and was performed in accordance with the principles of the Declaration of Helsinki.

### Statistical analysis

Statistical analysis was carried out using Python Pymer4, version 3.11.8. The level of significance was set to 0.05.

Descriptive statistical analysis includes *n*, mean, and standard deviation (SD) or median and interquartile range (IQR) in continuous variables, as well as percentages for categorical variables.

A mixed-effects logistic regression model was fitted to allow the analysis of random effects, such as individual patients and stimulation cycles, and the fixed effect of each embryo transfer on pregnancy and live birth rates. This model facilitates the analysis of hierarchical variables, to take into account the effect of each subsequent same-cohort embryo transfer in the order performed in each patient.

### Data collection

Fresh-cycle patient data included maternal age, body mass index, primary/secondary infertility, indication for fertility treatment, number of previous IVF/ICSI cycles, number of previous embryo transfers, baseline and follow-up hormonal status, type of stimulation protocol and drugs used, number of oocytes retrieved, oocyte quality, embryo progression, endometrial thickness, day of transfer, and number of fresh embryos transferred. Frozen-cycle data additionally included day of freezing, number of embryos thawed, number of thawed embryos transferred, and embryo quality.

Outcomes including positive serum βhCG 14 days after transfer, clinical and ongoing pregnancy rate, and live birth rate were ascertained.

## Results

### Patient characteristics

The characteristics of the patients are presented in Table 1. Patient characteristics are provided for the patient group with positive and negative βhCG after ET, respectively.

**Table 1:** Baseline characteristics of the study population

	Total patients (***n*** = 3635)	Positive βhCG after ET	Negative βhCG after ET	
Age (mean, SD)	33.7 ± 4.7	32.9 ± 4.6	34.3 ± 4.9	*P* < 0.001
Total no. of embryos transferred (*n*)	8118	1863	2930	n.s
Fresh embryo transfers (*n*, %)	4793 (59%)	1863	2930	
Frozen–thawed embryo transfers (*n*, %)	3325 (41%)	–	–	
No. of embryos per transfer (median, IQR)	1 (1) [(min, max) 1, 2]	1 (1) [(min, max) 1, 2]	1 (1) [(min, max) 1, 2]	
Peak estradiol	1583 ± 1115	1647 ± 1109	1542 ± 1117	*P* < 0.001
nCOC	9.0 ± 5.3	9.8 ± 5.3	8.5 ± 5.2	*P* < 0.001
MII	7.1 ± 4.3	7.7 ± 4.4	6.6 ± 4.2	*P* < 0.001
Method of fertilization	(*n* = 6284)			
IVF (*n*, %)	281 (4.5%)	82 (4.4%)	139 (4.7%)	n.s
ICSI (*n*, %)	6003 (95.5%)	1781 (95.6%)	2791 (95.3%)	n.s
No. of stimulations per patient (mean, SD)	1.6 ± 1	–	–	
No. of embryo transfers per stimulation (mean, SD)	1.6 ± 1	–	–	
SET (*n*, %)	6957 (85.7%)	1458 (78.3%)	2350 (80.2%)	n.s
DET (*n*, %)	1161 (14.3%)	405 (21.7%)	580 (19.8%)	n.s
Type of infertility (*n*, %)				
Primary infertility	2762 (58.5%)	1088 (59.5%)	1674 (58.7%)	n.s
Secondary infertility	1956 (41.5%)	742 (40.5%)	1177 (41.3%)	n.s
Etiology of infertility (*n*, %)				
Male factor	3102 (66.2%)	1236 (77.8%)	1881 (78.4%)	n.s
Endometriosis	585 (12.5%)	235 (14.8%)	351 (14.6%)	n.s
Tubal factor	475 (10.1%)	168 (10.6%)	311 (13.0%)	*P* = 0.025
PCOS	365 (7.8%)	182 (11.5%)	183 (7.6%)	*P* < 0.001
Other	159 (3.4%)	84 (5.3%)	141 (5.9%)	n.s

*DET* double embryo transfer, *ICSI* intracytoplasmic sperm injection, *IQR* interquartile range, *IVF* in vitro fertilization, *PCOS* polycystic ovary syndrome, *SD* standard deviation, *SET* single embryo transfer

During the study period, a total of 9719 fresh and frozen–thawed embryo transfers were carried out at the study center. After exclusion criteria had been applied, 8118 fresh and frozen transfers performed in 3635 patients were eligible for analysis. Each patient underwent at least one fresh embryo transfer and subsequently a various number of vitrified-warmed embryo transfers. Out of all embryo transfers analyzed, 59% were fresh embryo transfers and 41% were frozen embryo transfers. Patients with positive βhCG after ET were younger than those with negative βhCG after ET and had a higher oocyte yield. As indicated in Table 1, ICSI was performed in most cases. A single embryo transfer (SET) was carried out in the majority of cases, with the highest proportion of SETs in the fresh embryo transfer group. With an increasing number of subsequent vitrified–warmed cycles, there was a tendency towards an increase in double embryo transfers.

The etiology of infertility ranged from male factor in 66.2%, endometriosis in 12.5%, tubal factor in 10.1%, to polycystic ovarian syndrome (PCOS) in 7.8%. In many cases, combined etiology of infertility was documented. Tubal factor was found more often in the patient group with negative βhCG after ET, while PCOS was found more often in the group with positive βhCG after ET.

### Pregnancy rates after fresh and frozen embryo transfers

Pregnancy rates after fresh and frozen embryo transfers were calculated, and FET outcomes were analyzed separately for positive and negative βhCG after previous ET. The overall pregnancy rate after ET in this group of patients was 38.7%. In women ≤ 35 years of age and in women > 35 years, the pregnancy rates after ET were 43.3% and 31.7%, respectively.

In all patients with positive serum βhCG 14 days after ET, the model calculated a 36.3% [33.2%, 39.5%] probability of pregnancy after the subsequent FET. Positive βhCG after ET was found to have an effect on the subsequent FET outcome (*P* = 0.002). In patients with negative βhCG after ET, the pregnancy rate was 33.1% [30.7%, 35.7%] after the subsequent FET, regardless of patient age at FET. After the application of the mixed-effects logistic regression model, negative ET did not predict the outcome of subsequent FET (*P* = 0.726).

The pregnancy rate in the second same-cohort FET following a positive FET was 29.2% [23.9%, 35.2%] according to the model. A positive serum βhCG after the first FET could not be used to predict the outcome of subsequent frozen embryo transfers (*P* = 0.055). When the first FET from one cohort was negative, the pregnancy rate of the subsequent FET was 31.6% [28.2%, 35.5%] when an embryo from the same cohort was used. In this case, the model could not predict the outcome of subsequent frozen embryo transfers (*P* = 0.925).

### Clinical pregnancy rates after fresh and frozen embryo transfers

The clinical pregnancy rates (CPR) after fresh and frozen embryo transfers were calculated, and FET outcomes were again analyzed separately relative to previous ETs with and without a clinical pregnancy. The CPR after ET was 29.5% in all age groups. In women ≤ 35 years and > 35 years of age, the CPRs were 36% and 25.9%, respectively.

According to the mixed-effects model used, women with a clinical pregnancy after fresh transfer had a chance of 25.4% [21.6%, 29.5%] for a clinical pregnancy after the following FET. A clinical pregnancy in the fresh cycle was able to predict the chances of a clinical pregnancy after the first frozen embryo transfer from the same cohort (*P* = 0.009).

Following an unsuccessful ET, the clinical pregnancy rate after subsequent FET was 24.9% [22.4%, 27.5%].

### Live birth rates after fresh and frozen embryo transfers

Live birth rates (LBR) after fresh and frozen embryo transfers were analyzed, and FET outcomes in women who had a live birth after ET were compared with the outcomes in those who did not. Live birth rates after ET were 27.4% in total. The LBR was 31.8% in women ≤ 35 years and 20.4% in women > 35 years of age.

According to the mixed-effects model, women who had a live birth after a fresh embryo transfer had a live birth rate of 21.9% [18.9%, 25.3%] after the first same-cohort FET. The model confirmed that a live birth after fresh ET was a predictor for the FET outcome (*P* < 0.001).

In women in whom fresh ET did not result in a live birth, there was a live birth rate of 21.5% [19.6%, 23.5%] after the subsequent same-cohort vitrified–warmed cycle.

### Pregnancy and cumulative live birth rates after fresh and multiple subsequent vitrified–warmed embryo transfers

A mixed-effects logistic regression model was fitted to calculate pregnancy rates after the transfer of one fresh embryo and subsequently one, two, or more vitrified–warmed embryos from the same cohort. The model predicted an overall pregnancy rate of 34.8% after fresh embryo transfer, independent of patient age. The probability of pregnancy after the first vitrified–warmed embryo transfer was 32.8%. The second FET had a pregnancy rate of 30.8%, the third one 28.9%, and the fourth 27.1%. Further probabilities of pregnancy were calculated by the mixed-effects logistic regression model. Regardless of the outcome of the preceding embryo transfers, the model predicted continuously high chances of pregnancy after multiple consecutive same-cohort embryo transfers, with a hypothetical pregnancy rate of 23.7% after the sixth FET from the same cohort.

**Fig. 1:**
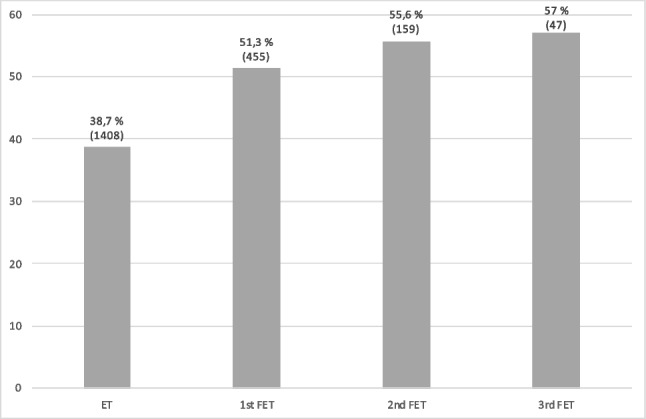
Cumulative live birth rates in the study cohort

Cumulative live birth rates in the cohort were analyzed after subsequent fresh and vitrified–warmed embryo transfers. These results are depicted in Fig. 1.

A cumulative live birth rate of 57% was observed after four subsequent fresh and vitrified–warmed embryo transfers.

## Discussion

These findings clearly show that the fresh transfer outcome has an effect on the subsequent first frozen–thawed embryo transfer. A total of 8118 fresh and frozen cycles were included in the study, providing robust data. To the best of our knowledge, this is the largest clinical study that has assessed the effect of fresh transfer outcomes on subsequent frozen embryo transfers, and it is the first study taking into account multiple consecutive vitrified–warmed embryo transfers and live birth rates. A positive serum βhCG 14 days after fresh embryo transfer has a significant effect on pregnancy rates in the subsequent FET. Women with positive βhCG after ET were younger and had a higher oocyte yield per stimulation than women with a negative βhCG after ET. These findings are in accordance with previously published data [21, 32, 33]. However, we found that an unsuccessful fresh cycle is not predictive for subsequent FET pregnancy rates. Even though these women were older and had a lower oocyte yield per stimulation, negative βhCG after ET did not have an effect on their FET outcomes. Pregnancy rates after FET were higher after a successful ET but tended to be lower after an unsuccessful ET, although without any statistical significance. This supports the hypothesis that good embryo quality will result in high chances of pregnancy in both fresh and frozen cycles.

Adjustment for embryo quality was not carried out during the analysis, since only high-quality embryos were used in all cases. The embryo stage on the day of cryopreservation was not included in the analysis. However, previous studies have shown that the day of freezing does not affect FET pregnancy rates [23, 34]. In contrast to other studies [7, 19, 22, 25], vitrification was the cryopreservation technique used in the present cohort, to ensure adherence to modern standards in ART.

Mixed-effects logistic regression analysis was carried out to incorporate both fixed and random effects and to account for the nested associations caused by subsequent embryo transfers in the sequence in which they were performed. The effect of each subsequent embryo transfer was fitted as the fixed effect. Random effects were added to account for the statistical variability caused by controlled ovarian stimulation cycles or by individual patients.

Medical documentation has been growing in importance in recent years, with the goal of collecting high-quality data for research purposes. As the period covered by the present study spans more than a decade, there have been substantial changes in the amount, quality, and type of data collected. Some parameters in the dataset have not always been documented in the early years of the study, such as type and etiology of infertility. This explains isolated missing data points in the dataset, which are compensated for by the large number of cases overall.

Comparison of the findings with previous studies shows that Bushaqer et al., for example, reported similar results, with higher pregnancy rates in FET after successful fresh ET. However, only a small number of frozen–thawed transfers were included in that study (*n* = 84), and there was an age difference between the fresh cycle group, in which ET was only offered to women younger than 35 years of age, and the FET group, in which women were included regardless of their age [23].

The present findings contradict the results reported by Doherty et al., which suggested that frozen–thawed ET is more likely to succeed after an unsuccessful fresh ET using embryos from the same cohort. Only cryopreserved embryos using the slow-freezing method were included in the study. Doherty’s research group claims that only a small number of blastocysts resulting from one ovarian stimulation are capable of resulting in a viable pregnancy and that clinicians do not always choose the right embryo for ET [25]. In contrast to the paper by Doherty et al., mixed-effects logistic regression was used in the present study to rule out possible effects caused by individual patients and stimulation numbers, and the study included only vitrified embryos. The model showed continuously high pregnancy rates after consecutive transfer of cryopreserved embryos. According to the model, the chances of pregnancy after subsequent same-cohort embryo transfers remain excellent when the pregnancy rates of fresh cycles are compared with the pregnancy rates of the sixth frozen embryo transfer, at 34.8% and 23.7%, respectively. The cumulative live birth rate was 57% after four consecutive transfers.

Zargar et al. reported significantly higher pregnancy rates after FET in comparison with fresh ET in general and higher pregnancy rates after successful ET in comparison with previous unsuccessful ET [24]. However, fewer than 700 patients were included in the study. Li et al. observed a higher live birth rate after FET in women with a previous first-trimester miscarriage following FET (Li et al., 2020). Although only freeze-all protocols were carried out in the study, the authors found that a positive serum βhCG 14 days after embryo transfer was a good prognostic factor for subsequent frozen embryo transfers.

In the present study, a tendency towards lower pregnancy rates was detected in FET after an unsuccessful fresh cycle (33.1% vs. 36.3%). There has been a trend in recent years towards elective freezing to eliminate possible negative effects of controlled ovarian stimulation on endometrial receptivity and to prevent ovarian hyperstimulation syndrome (OHSS). Using a freeze-all strategy is not recommended for all patient groups [8, 17, 35]. The present data support this recommendation. However, in some situations, elective freeze-all cycles are the regimen of choice and should be carefully discussed with couples. Indications include planned preimplantation genetic testing, endometrial pathology, or OHSS [17, 36]. Higher pregnancy rates were not found after FET in general. Endometrial receptivity cannot therefore be solely dependent on controlled ovarian stimulation, but must also be affected by intrinsic factors [37–39].

The question of whether to use cryopreserved embryos or to initiate a fresh stimulation cycle is often a matter of difficult discussion with patients. In Austria and other countries, only a limited number of IVF/ICSI attempts are partly covered by public health insurance, and this affects the decision on which procedure to choose [40]. The women’s age also plays a role in the decision-making in many cases, as financial support by insurance companies for fertility treatments ceases as soon as the patient reaches a certain age. Furthermore, the number of embryos cryopreserved, results of preimplantation genetic testing, and each couple’s individual family planning wishes need to be taken into consideration. Couples who wish for more children or women of advanced age might have a tendency to ask for another stimulation cycle than to make use of previously cryopreserved embryos. Women who only have one single embryo cryopreserved will also be more likely to choose another ovarian stimulation cycle than those patients who still have several embryos cryopreserved.

## Conclusion

To conclude, this study shows that positive serum βhCG, ultrasound confirmation of a fetal heartbeat, and live birth after fresh embryo transfer are associated with a higher probability of pregnancy in the subsequent same-cohort frozen–thawed cycle. An unsuccessful fresh cycle cannot be used to predict the subsequent same-cohort frozen embryo transfer outcome.

Pregnancy rates only decline gradually after subsequent transfers of cryopreserved embryos, with a pregnancy rate of over 20% after the sixth vitrified–warmed embryo transfer. The cumulative live birth rate in our cohort was high, at 57% after the third frozen cycle.

In the past, robust data have not been available to provide these patients with information about their individual chances for a pregnancy. As health care is becoming increasingly individualized, the present findings may help provide an evidence base for counseling couples, taking their individual fertility history into account. Our prediction model aids to provide both evidence-based and individualized counseling of patients with infertility.

## Data Availability

The datasets used and/or analyzed during the current study are available from the corresponding author on reasonable request.

## Abbreviations

ART: Assisted reproductive technology
βhCG: Beta human chorionic gonadotropin
CPR: Clinical pregnancy rate
DET: Double embryo transfer
ET: Fresh embryo transfer
FET: Frozen embryo transfer
ICSI: Intracytoplasmic sperm injection
IQR: Interquartile range
IVF: In vitro fertilization
LBR: Live birth rate
PCOS: Polycystic ovary syndrome
SD: Standard deviation
SET: Single embryo transfer
